# Wearable-Sensor-Based Weakly Supervised Parkinson’s Disease Assessment with Data Augmentation

**DOI:** 10.3390/s24041196

**Published:** 2024-02-12

**Authors:** Peng Yue, Ziheng Li, Menghui Zhou, Xulong Wang, Po Yang

**Affiliations:** 1 Department of Computer Science, University of Sheffield, Sheffield S10 2TN, UK; pyue1@sheffield.ac.uk (P.Y.); menghuizhoucn@gmail.com (M.Z.); xl.wang@sheffield.ac.uk (X.W.); 2 AntData Ltd., Liverpool L16 2AE, UK; 3 Department of Software, Yunnan University, Kunming 650106, China; liziheng9050@mail.ynu.edu.cn

**Keywords:** Parkinson’s disease, activity recognition, wearable sensor, weak annotation, class imbalance, data augmentation

## Abstract

Parkinson’s disease (PD) is the second most prevalent dementia in the world. Wearable technology has been useful in the computer-aided diagnosis and long-term monitoring of PD in recent years. The fundamental issue remains how to assess the severity of PD using wearable devices in an efficient and accurate manner. However, in the real-world free-living environment, there are two difficult issues, poor annotation and class imbalance, both of which could potentially impede the automatic assessment of PD. To address these challenges, we propose a novel framework for assessing the severity of PD patient’s in a free-living environment. Specifically, we use clustering methods to learn latent categories from the same activities, while latent Dirichlet allocation (LDA) topic models are utilized to capture latent features from multiple activities. Then, to mitigate the impact of data imbalance, we augment bag-level data while retaining key instance prototypes. To comprehensively demonstrate the efficacy of our proposed framework, we collected a dataset containing wearable-sensor signals from 83 individuals in real-life free-living conditions. The experimental results show that our framework achieves an astounding 73.48% accuracy in the fine-grained (normal, mild, moderate, severe) classification of PD severity based on hand movements. Overall, this study contributes to more accurate PD self-diagnosis in the wild, allowing doctors to provide remote drug intervention guidance.

## 1. Introduction

Parkinson’s disease (PD) is the second most common dementia in the world, affecting a significant proportion of the elderly population [1]. According to [2], approximately 9 million people in the ten most populous countries will suffer from this disease by 2030. PD is characterized by a severe loss of dopamine in the forebrain, resulting in motor symptoms such as tremors, muscle stiffness, bradykinesia, postural instability, as well as non-motor symptoms including hyposmia, sleep disturbances, and autonomic dysfunction [3]. The disability rate among PD patients is notably high [4]. Furthermore, there is currently no cure for PD. All drug treatments can only relieve symptoms, reduce complications, and prolong life. Although Parkinson’s disease is incurable, a quantitative assessment of PD symptoms is necessary because it can help doctors use appropriate targeted interventions. To effectively assess these motor symptoms, rating scales have been widely adopted, such as the MDS-Unified Parkinson’s Disease Rating Scale (MDS-UPDRS) [5]. However, these assessments typically occur in clinical settings with infrequent annual visits, and the MDS-UPDRS evaluation is time-consuming, requiring at least 30 min and specialized training [6]. These factors all contribute to the difficulty in effectively monitoring PD. As a result, convenient and objective PD assessment tools are required to better assist patients.

With the widespread adoption of wearable devices and the advancements in machine learning technology [7,8], there has been a significant body of research dedicated to the objective assessment of PD symptom severity using wearable inertial sensors. For instance, ref. [9] used a wearable device on the hand to detect the number of finger-taps to assess the severity of bradykinesia, ref. [10] developed a CNN-LSTM network to detect PD gait freezing using pressure sensors in insoles, and [11] used two IMU sensors on the wrist and then asked subjects to perform 11 tasks to detect the early stages of PD. These works demonstrate the great potential of using wearable devices to assess PD.

Despite the significant promise demonstrated by wearable technology in monitoring PD symptoms, the development of a system and algorithm for assessing the disease stage of PD patients remains a challenging endeavor. Previous studies have predominantly concentrated on the detection of individual symptoms, such as tremor [12], bradykinesia [13,14], and gait freezing [15]. While these studies have effectively gauged the severity of specific PD symptoms, they fall short in providing a comprehensive evaluation of the overall disease stage of PD patients.

**Figure 1:**
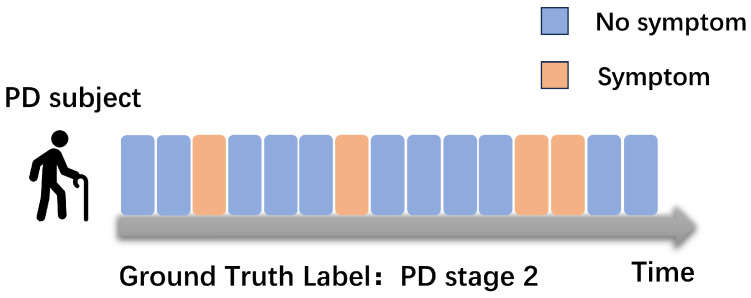
For the problem of inaccurate labeling in weak supervision, in most cases we can only obtain ground-truth coarse-grained PD-stage labels, and it is unknown which segments in the signal have PD symptoms.

The clinical scale MDS-UPDRS evaluates the severity of PD based on symptoms manifested during various activities. Previous research has been limited to a single symptom and activity. To achieve a more comprehensive assessment of PD severity, it is imperative to consider the severity of multiple symptoms. However, real-world scenarios present the following two crucial challenges:

- Limited annotation: In practical scenarios, obtaining detailed symptom annotations is a time-consuming endeavor [16]. Especially in a free-living environment, the workload of obtaining accurate annotations of when PD symptoms begin and end is huge. PD symptoms are intermittent, which means that PD symptoms may be sparsely distributed and their time of appearance is unpredictable. However, we can only obtain coarse-grained annotations of PD stages, which makes training a supervised classifier to evaluate PD stages difficult. In fact, most current approaches are to cut the signal into shorter time segments, and then assign the overall disease severity label to each segment. However, this approach introduces a lot of noise segments, especially when symptoms are sparsely distributed. Figure 1 shows the weak-label problem. This situation is commonly recognized as a weakly supervised problem, which has motivated us to develop a recognition framework within the context of weak supervision.
- Class imbalance: The majority of patients tend to fall into the category of mild PD, with a relatively small proportion classified as severe (in our collected dataset, there was only about a quarter as many severe patients as mild patients). This results in a class imbalance issue. Additionally, there is often substantial variability in motor performance among PD patients at the same disease stage. Hence, we must explore effective strategies for leveraging our data to address this challenge.

The factors mentioned above present formidable challenges when it comes to assessing the stage of PD patients. Leveraging these challenges as a foundation, we introduce an innovative framework for PD diagnosis under weakly supervised conditions, with the goal of addressing the PD diagnostic problem in natural and daily environments characterized by limited annotation and data imbalance.

Our approach comprises two key components. First, we establish a PD learning framework under weak supervision. Initially, we employ fixed-window segments to extract features from the sensor signal data generated during various activities by PD patients. Subsequently, we apply k-means clustering to group together segments from the same activity across all patients. To uncover latent associations among different activities of PD patients, we utilize latent Dirichlet allocation (LDA) topic models to generate global features from these clustering labels. These two sets of features are then fused to create a refined representation of the PD patient features. Additionally, we introduce a data augmentation technique that identifies similar patient pairs through similarity comparisons and mixes them. Simultaneously, we disrupt the sequence of different segments within the data to reduce the reliance on segment position and time, thereby generating more diverse samples. These generated pseudo-data samples are used for training. Our approach is grounded in the belief that fine-grained features extracted from short-term fixed windows may not adequately capture the overall disease stage of a patient. Thus, we aim to unveil implicit associations between various activities of patients through unsupervised topic modeling. Furthermore, our data augmentation method enriches feature expressions while maintaining semantic consistency. Finally, we conduct a PD-stage classification test using a real-world dataset comprising 83 individuals in a free-living environment. Our approach achieves an accuracy rate of 73.48% in classifying PD stages (normal, mild, moderate, severe), surpassing segment-based PD-stage classification by 17%. This outcome serves as compelling evidence of the effectiveness of our method.

In summary, the main contributions of this work can be outlined as follows:

- We propose a framework that utilizes the idea of multi-instance learning to combine symptom representations from multiple different activities to assess PD severity.
- We propose a novel framework to accurately assess the status of PD patients within a weakly annotated context. We first combine local features from multiple segments with global topic features from various activities to perform classification. To address the problem of class imbalance, we present a straightforward yet highly effective data augmentation technique designed to generate additional data, enriching the original dataset, and enhancing the classification performance, particularly for minority classes.
- To fully demonstrate the efficacy of our proposed framework, we collected a dataset containing wearable-sensor signals from 83 individuals in real-life, free-living conditions. Not only that, but this dataset also contains comprehensive and diverse wearable-sensor signals of a total of 12 human activities from each individual, which has never been provided in any previous works (to the best of our knowledge).
- The detailed experimental results shows that our framework achieves an astounding 73.48% accuracy in the fine-grained (normal, mild, moderate, severe) classification of PD severity based on hand movements, which verifies the feasibility of accurately identifying PD patients based on machine learning and wearable-sensor data.

The remainder of this paper is structured as follows: Section 2 delves into the relevant literature, while Section 3 covers data collection and data preprocessing. In Section 4, we introduce our framework for PD-stage assessment, followed by an evaluation and presentation of the results in Section 5. Finally, Section 6 provides the concluding remarks for this paper.

## 2. Related Work

We examine the related work from three perspectives: (1) assessing PD severity using wearable technology, (2) weakly supervised learning, and (3) augmentation of time-series data.

### 2.1. Wearable Technology for PD-Severity Assessment

Numerous studies have investigated the monitoring and management of PD motor symptoms using wearable technology, demonstrating its effectiveness comparable to clinical scores [17]. These studies employ single or multiple wearable devices to quantify or chronically manage PD. Currently, the focus of PD monitoring primarily includes the following four symptoms: (1) bradykinesia [13], (2) tremor [12], (3) gait freezing [15], and (4) muscle rigidity [18]. These studies have shown promising results in monitoring individual PD motor symptoms using wearable technology.

However, most of the existing studies focus on individual symptoms. This is because the comprehensive evaluation of multiple PD motor symptoms, typically required for PD staging, presents challenges. Nevertheless, recent research has started considering evaluations involving multiple activities. For instance, Aleksandr Talitckii et al. [19] found that considering both tremor and bradycardia symptoms significantly improved the accuracy of distinguishing PD from healthy individuals. Luis Sigcha et al. [20] proposed an evaluation method called session base, where they asked patients to perform finger tapping. Existing methods often segment data using fixed windows and rely on fine-grained symptom labels. These PD evaluation studies do not take into account the weak-labeling problem, which may lead to degraded classification performance in weakly labeled free-living environments.

### 2.2. Weakly Supervised Learning

In practical scenarios, it can be challenging for PD patients to accurately record the onset time of each symptom. However, PD-stage labels based on long-term observation are available, often considered within a weakly supervised learning setting [21]. To tackle this issue, numerous studies employ multiple-instance learning (MIL) methods. Existing MIL methods can be broadly categorized into two types: instance-level methods and bag-level methods. In the instance-level assumption, the contribution of all instances is considered equal and is aggregated, often using methods like voting aggregation [22]. However, this approach is susceptible to incorrect instances and struggles to obtain instance labels [23]. In contrast, bag-level methods typically consider the information from multiple instances and often yield higher accuracy [22]. While MIL has found widespread use in various fields, including medicine [24], and bioinformatics [25], it has seen limited application in human activity recognition and PD evaluation [26]. The study in [27] used tremor time proportion labels to reduce the impact of long-term weakly labeled data on Parkinson’s tremor classification. However, this method uses more detailed labels. We believe it is impossible to obtain such detailed annotation in a free-living environment. Hence, in this study, we propose an MIL-based framework for PD diagnosis to address the weak-annotation challenge encountered in real-world scenarios.

### 2.3. Time-Series Data Augmentation

Data augmentation serves as an effective method to expand the number and diversity of samples when dealing with limited data, especially in the domain of medical data, where data collection can be costly and class imbalances are common. One approach involves generating more diverse samples through slight transformations in the original signal to enhance feature representation, as demonstrated in [28]. These methods include permutation, magnitude-warping, cropping, jittering, and rotation, leading to improvements in classifying PD bradykinesia and dyskinesia states. However, this approach may not be suitable for PD-stage evaluation since magnitude-warping and jittering can alter symptom severity and disregard inter-patient differences. Additionally, there are data augmentation methods based on the frequency domain, such as those utilizing empirical mode decomposition (EMD) [29]. These methods decompose signals into different intrinsic mode functions (IMFs) using EMD and recombine them to diversify the data. The above-mentioned work often uses a single timing signal for data augmentation, while ignoring the correlation between different signal axes. In fact, there are often multiple different channel signals in the sensor signal. In our study, we introduce a framework-compliant data augmentation method that retains information on multiple PD activities and mixes data from various patients to generate richer samples.

## 3. Data Collection

The data utilized in this research study were gathered by our team at the hospital over the period spanning from 15 January 2021 to 30 July 2022. The study involved 70 individuals diagnosed with PD and 15 healthy volunteers who willingly participated. Every participant provided informed consent. The total of 85 individuals can be divided into four categories, namely, healthy people, mild-PD patients (mild), moderate-PD patients (moderate), and severe-PD patients (severe). Please refer to Table 1 for detailed demographics of this dataset.

**Figure 2:**
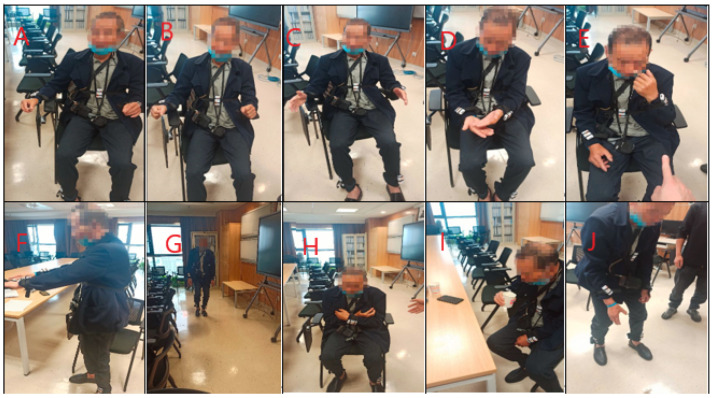
Overview of activities: (**A**) Finger taps. (**B**) Clenching and opening alternately. (**C**) Rapid alternating movements of hands. (**D**) Hand rotation—right/left. (**E**) Finger to nose—left/right. (**F**) Standing with arms held out. (**G**) Walking back and forth. (**H**) Rising from chair. (**I**) Drinking water. (**J**) Picking things up.

Throughout the study, participants were equipped with Shimmer3 inertial measurement units (Dublin) (IMUs) on various body parts, including the left wrist, right wrist, left ankle, right ankle, and waist, for the collection of acceleration and gyroscope signal data. In subsequent experiments, data solely from the right wrist sensor were utilized to ease the burden on patients. The Shimmer3 IMU communicated wirelessly with a computer via Bluetooth. The ConsensysPRO software (Ver1.5.0) on the computer was employed to collect signal data at a high sampling frequency of 200 Hz. Each participant engaged in 12 distinct activities, with a 1-min rest interval between each activity. Prior to commencing the experiments, researchers provided instructions to the PD patients regarding the activity requirements. Once the experiments were underway, no further guidance or interference from the investigators was provided. Video recordings were made during the data collection process, and a neurologist subsequently scored all the data according to the Hoehn–Yahr (H –Y) scale, considering the participant’s performance across multiple activities. Individual-level labels were assigned based on this assessment. Table 2 and Figure 2 present a comprehensive list of the activities performed during the experimental setup. After collecting the data, we excluded patients whose activity lasted less than 20 s, and finally 83 subjects met the requirements.

## 4. Methodology

### 4.1. Framework Overview

**Figure 3:**
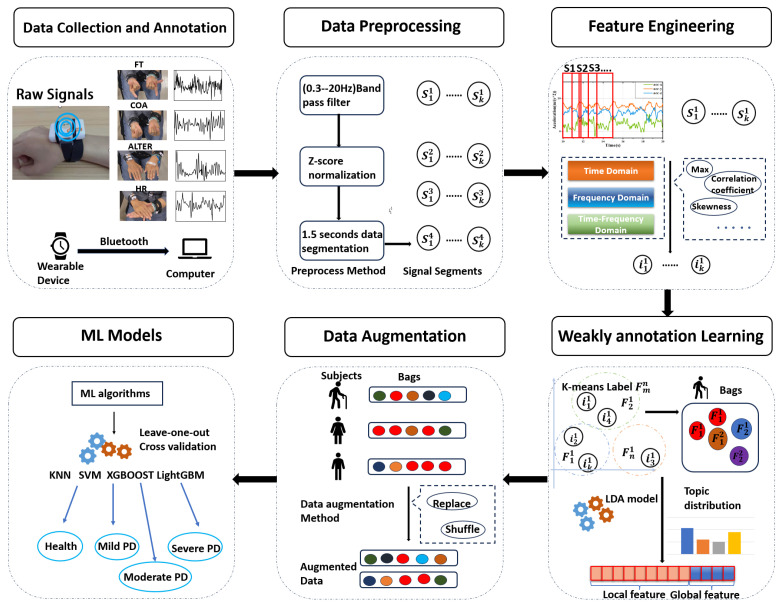
Overview of the framework: The original wearable device signal is decomposed into various segments via slicing and feature extraction, and these segments are clustered to generate cluster labels. The document is composed of multiple active clustering labels, and the topic probability distribution is generated by the topic model as the extracted global features for classification. We then use the bag’s data augmentation method to generate more samples.

The comprehensive framework is visually represented in Figure 3. This framework is organized into five distinct components. The initial part encompasses data preprocessing and sliding window segmentation. The second component extracts the features from the original signal. In the third part, these fragment features are used for k-means clustering and aggregation. We then fit the distribution of these cluster labels through the LDA model to generate new global features. In the fourth part, we use data augmentation methods to generate more data and alleviate the negative effects of data imbalance. The final segment pertains to the training and testing phase, where machine learning models are trained and assessed using these features. This approach aims to develop a machine learning model which is capable of evaluating the disease severity of PD patients. Detailed descriptions of each step follow in the subsequent sections.

### 4.2. Problem Statement

We formulate the research problem as a four-class classification task. Given that the input features *x* represent the activities of participants, the objective of the model is to predict the PD stage *y*, which can fall into one of four categories: healthy, mild, moderate, or severe PD. This problem, distinct from binary classification between PD patients and non-PD patients, presents a much greater challenge due to the potential similarity in features exhibited by PD patients at various stages during specific time intervals.

To solve the problem of inaccurate supervision, we adopt the multiple-instance learning (MIL) method. In MIL, each learned sample is defined as a bag containing multiple instances. Different from traditional single-instance learning, each package contains the feature space of different instances. We define the bag collection $Bag=\{X_{1},X_{2},X_{3}\cdots X_{j}\}$; for each bag $X_{j}$ has *m* instances $\left\{x_{j1},x_{j2},x_{j3},\cdots ,x_{jm}\right\}$. Here, we adopt MIL’s bag assumption: each instance is independent of the label, and the bag is related to the label. The classification tasks occur at the bag level.

### 4.3. Data Preprocessing and Segmentation

**Figure 4:**
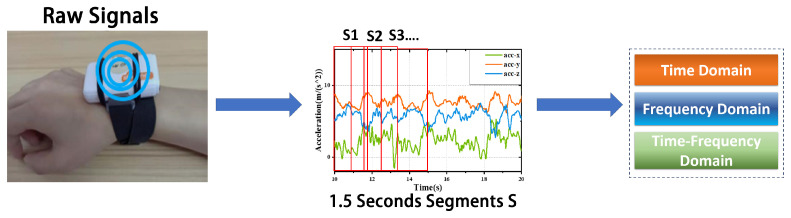
Data preprocessing and segmentation.

Tremor in PD patients can be classified into three types: rest tremor, 3–6 Hz; postural tremor, 4–12 Hz; and kinetic tremor, 2–7 Hz [30]. To smooth the signal and remove the gravity component, we use a 4th-order Butterworth filter with a bandpass range of 0.3–20 Hz. After applying Z-score normalization to the signal, the data are sliced at 300 data points (1.5 s) with a 50% overlap. Finally, for each window signal, we compute the time- and frequency-domain-related features (standard deviation, variance, skewness, kurtosis, root mean square, energy, median, range, correlation). The preprocessing method is depicted in Figure 4.

### 4.4. Feature Extraction and Fusion

- **Clustering for Document Creation:** As depicted in Figure 3, the activity signals performed by the patient are segmented into segments represented as
  $$[i_{1}^{1},i_{2}^{1},i_{3}^{1},i_{4}^{1},\cdots ,i_{1}^{2},i_{2}^{2},i_{3}^{2},i_{4}^{2},\cdots i_{n}^{m}],$$
  where *m* denotes the *m*-th activity, *n* represents the *n*-th window segment of the activity, and *i* signifies the feature set of the segment. Subsequently, the feature sets of the same activity segment from different patients are clustered using k-means, and the resulting clustered labels are employed as words to construct documents:
  (1) $${\mathrm{Document}}_{p}=\left[F_{1}^{1},F_{1}^{1},\ldots ,F_{a}^{b}\right].$$
  Here, $F_{a}$ represents the k-means clustering label, *b* denotes the *b*-th activity, and *p* indicates the *p*-th subject. This method involves aggregating words from multiple patient activities to generate a document, followed by utilizing a topic model to derive global features.
- **LDA Topic Model for Global Feature Generation:** In our framework, latent Dirichlet allocation (LDA) [31] is utilized to discover global topic features across various activities. The document–word matrix serves as input to LDA, which subsequently outputs the document–topic distribution as global features. The document–topic distribution is defined as

(2) $$\begin{array}{cc} P(W,Z,\theta ,\varphi ;\alpha ,\beta )= & \prod_{j=1}^{M}P\left(\theta_{j};\alpha \right)\prod_{i=1}^{K}P\left(\varphi_{i};\beta \right)\\ \; & \prod_{t=1}^{N}P\left(Z_{j,t}|\theta_{j}\right)P\left(W_{j,t}|\varphi Z_{j,t}\right). \end{array}$$

**Figure 5:**
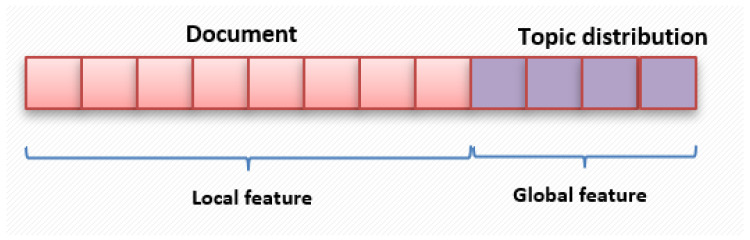
Feature vector.

The variables α and β represent Dirichlet distributions, θ signifies the topic distribution, and ϕ represents the word distribution. In the LDA model, a topic *z* is selected from the topic distribution θ, and a word *w* is chosen from the word distribution ϕ. A document comprises a collection of *N* words, while a corpus *D* consists of *M* documents, and *K* signifies the total number of topics in the corpus. LDA generates documents based on input parameters α, β, and *K*, governing the creation of topics and words [32]. As depicted in Figure 3, we input a document–word matrix into LDA, which produces a topic distribution. The probabilities associated with each topic are utilized as features. Ultimately, these feature vectors are combined horizontally to form the comprehensive set of features for PD recognition. This approach provides an advantage as each patient’s various activities generate a topic distribution feature, derived from the global information across multiple activities, resulting in richer information and enhanced feature expression. Figure 5 illustrates the final feature vector. Algorithm 1 demonstrates the entire process.
**Algorithm 1** Bag Generation for Multiple Activity Instances1:**function** Bag generation for multiple activity instances2:    **Input:** Instance $i_{n}^{m}$ of different activities3:    **Output:** Bag vector $O_{p}$4:    5:    **for** p←1 **to** Totalnumberofsubjects **do**6:        **for** instance $i_{n}^{m}$ **in** $subject_{p}$ **do**7:           $F_{a}^{b}$ = $kmeans(i_{n}^{m})$8:        **end for**9:        Create Document Feature ${Document}_{p}=\left[F_{1}^{1},F_{1}^{1},\ldots ,F_{a}^{b}\right]$10:      Create LDA Feature ${Topic}_{p}=LDA\left(Dcoument_{p}\right)$11:      $O_{p}=$ Horizontal binding vector $\left(Dcoument_{p},{Topic}_{p}\right)$12:  **end for**13:  14:  **Return** $O_{p}$15:**end function**

### 4.5. Data Augmentation

**Figure 6:**
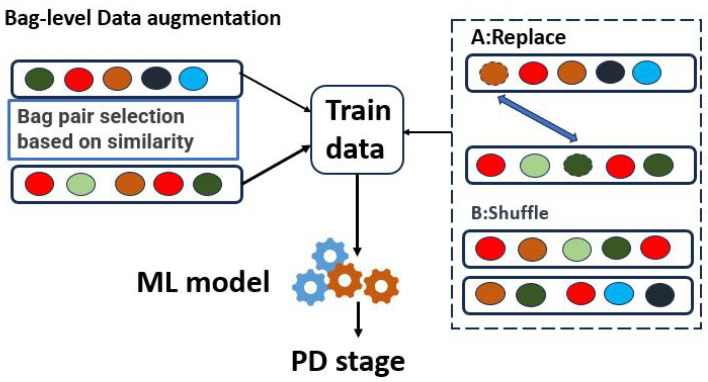
Bag-level augmentation method.

As mentioned in Section 1, data augmentation serves two primary purposes. Firstly, it aims to enhance the prediction accuracy of minority classes, addressing the issue of class imbalance. Secondly, it seeks to reduce variability between patients, thus improving the model’s robustness. Figure 6 illustrates the data augmentation method. Initially, we organize the instance clustering labels into vectors A and B according to their original chronological order. Subsequently, we compute the distances between different patients using the following (3) and select pairs with close distances:

(3) $$\mathrm{Distance}(H)=\sum_{i=1}^{n}\left(A_{i}\neq B_{i}\right)$$

We employ two methods for mixing the samples: ① We randomly shuffle similar sample pairs to generate new samples. These bag pairs originate from patients at the same PD stage, so the labels remain unchanged. This process increases sample diversity and mitigates intra-class differences. ② We shuffle the order of instances within each bag to address the uncertainty surrounding when patients exhibit symptoms. Through shuffling, we generate samples that are independent of time.

## 5. Experimental Results

In this section, we initially assess the effectiveness of our proposed framework using various basic machine learning algorithms and compare it with other augmentation methods to demonstrate the advantages of our framework in PD-stage evaluation. At the same time, we compare the results of a single-activity bag and the results of a combined-activity bag to prove the necessity of multi-activity combinations. Our problem belongs to the imprecise supervision problem in weak supervision. We also compare the bag-mapping method to solve this problem to prove the effectiveness of the method.

### 5.1. Experimental Setup and Evaluation Methods

We employed different basic machine learning algorithms to evaluate our proposed framework, including k-nearest neighbors (KNN), support vector machine (SVM), XGBoost, and LightGBM. We utilized leave-one-out cross-validation on our dataset of 83 individuals. The specific hyperparameter settings are detailed in Table 3. Finally, we used accuracy (ACC), precision (P), recall (R), and F1-score as evaluation metrics:

(4) $$\begin{array}{cc} \;\mathrm{Accuracy}\; & =\frac{TP+TN}{TP+TN+FP+FN}. \end{array}$$

(5) $$\begin{array}{cc} \;\mathrm{Precision}\; & =\frac{TP}{TP+FP}. \end{array}$$

(6) $$\begin{array}{cc} \;\mathrm{Recall}\; & =\frac{TP}{TP+FN}. \end{array}$$

(7) $$\begin{array}{cc} \mathrm{F1-score} & =\frac{2\times (\mathrm{Precision}\;\times \;\mathrm{Recall})}{\left(\mathrm{Precision}\;+\;\mathrm{Recall}\right)}. \end{array}$$

Here, true positive (TP) signifies the correct prediction of positive samples, while false positive (FP) represents a false positive prediction. False negative (FN) indicates a false negative prediction, and true negative (TN) corresponds to the correct prediction of a negative sample.

Multi-instance learning has always been an effective method to solve the imprecise supervision problem in weak supervision; however, few studies have considered the combination of multi-instance learning and PD evaluation. Many methods need to introduce additional annotation information, and do not consider the case of multi-classification. For the above reasons, we need a classifier-independent multi-classification multi-instance learning method. We use the following instance-selection-based MIL baseline approaches for comparison:

- MILWA: Propagates the bag label to all the instances inside the bag as the bag representation [33].
- MILES: Mapping instance features to package features via instance similarity measure [34].
- BOF: Uses the bag of features to map the subject’s instances to obtain the subject’s coding vector, and finally, classifies the coding vector [35].
- FV: We also compared the more robust Fisher vector encoding, which also encodes instances, and finally, classifies the encoded vectors [36].
- MILIBRT: This method utilizes the Hausdorff distance between bags to encode instances, and introduces a weight calculation method to better distinguish different categories of packages [37].
- MIBRV: Uses the extended Hausdorff distance to encode instances to reduce the impact of abnormal instances on the bag [38].
- SimpleMI: Use methods such as arithmetic mean to combine multiple instances for representation, or select representative instances for classification [39].

These experimental results can be seen in Section 5.

### 5.2. Weak Annotation

**Figure 7:**
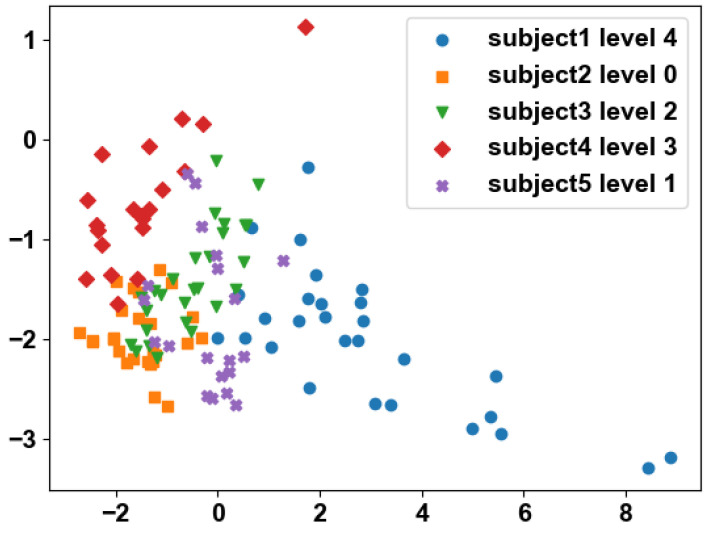
Data distribution after PCA dimension reduction. Each point represents the activity signal features of the subject in a 1.5 s window. Data distribution of different PD-severity levels.

Figure 7 illustrates the distribution of instances for five subjects at various stages of PD. It can be deduced that not all instances from PD patients are in the ON state, and there may be instances resembling level 0. However, obtaining instance-level labels poses challenges, leading to the emergence of the weak-labeling problem.

**Figure 8:**
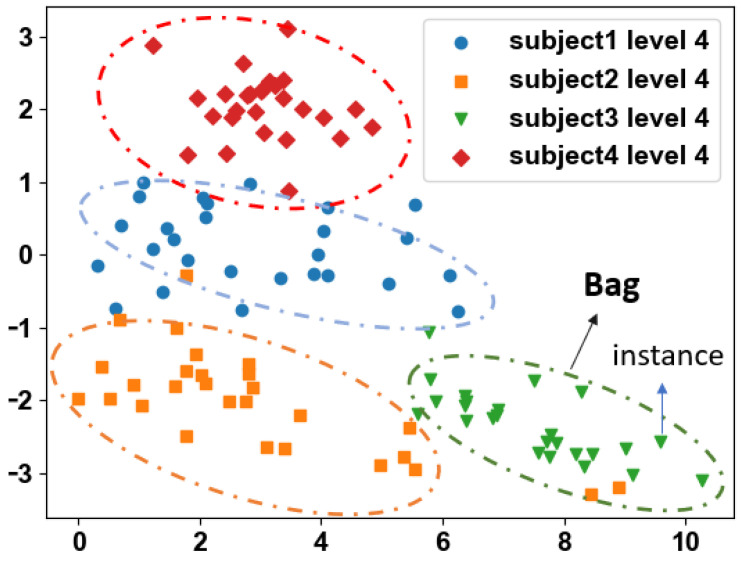
Data distribution after PCA dimension reduction. Each point represents the activity signal features of the subject in a 1.5 s window. Individual differences in the same category.

Figure 8 presents four subjects in PD-stage 4. Despite all of them performing the same activity and being labeled as PD-stage 4, differences in instance distributions are evident due to subject 1 developing tremors while subject 4 exhibited bradykinesia during the activity. This demonstrates that subjects within the same category may exhibit individual variations, making classification more challenging. Consequently, a larger and more diverse dataset is required to address these differences and enhance sample diversity.

### 5.3. Multi-Activity Bag Generation

Most previous work focuses on a single activity to assess PD. In practice, we found that a bag composed of multiple activity instances may be more conducive to the assessment of PD patients’ conditions. In Table 4, we compared the accuracy when a bag consists of a single activity instance and when a bag contains different combinations of activity instances. We found that adding a variety of different activities increases the accuracy. In a single activity, activities 2, 3, 4, 5, 7, and 8 achieved higher accuracy. It is speculated that the reason for this is that different subjects have different symptoms when performing different activities, and a single activity is not comprehensive for PD assessment. At the same time, in Table 5, we excluded the combination of leg activities because we found that a considerable number of severe-PD subjects were unable to perform this activity or had difficulty performing it autonomously, which may not be conducive to patients’ self-assessment at home. Table 5 shows the PD-evaluation accuracy of different activity combinations. It can be seen that the accuracy of the combination of activities is higher than that of a single activity. We select the combinations 2, 3, 4, and 5 with the highest average accuracy for the next experiment, and use the accuracy experiment with XGBoost, the base classifier with the highest rate.

### 5.4. Parameter Setting

**Figure 9:**
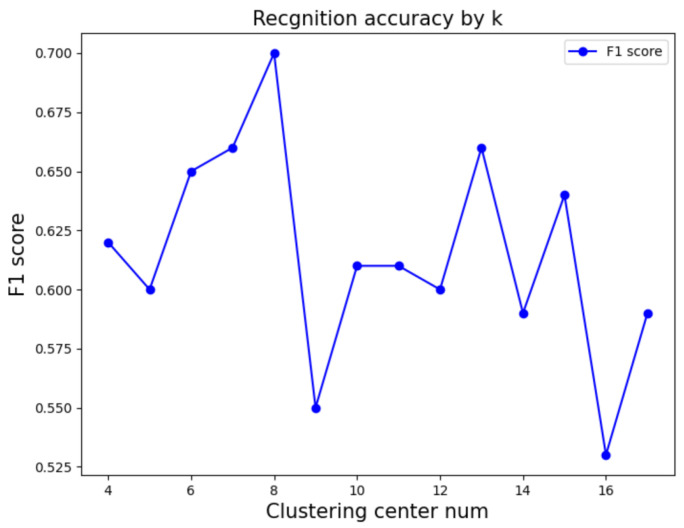
Parameter tuning of number of cluster centers.

**Figure 10:**
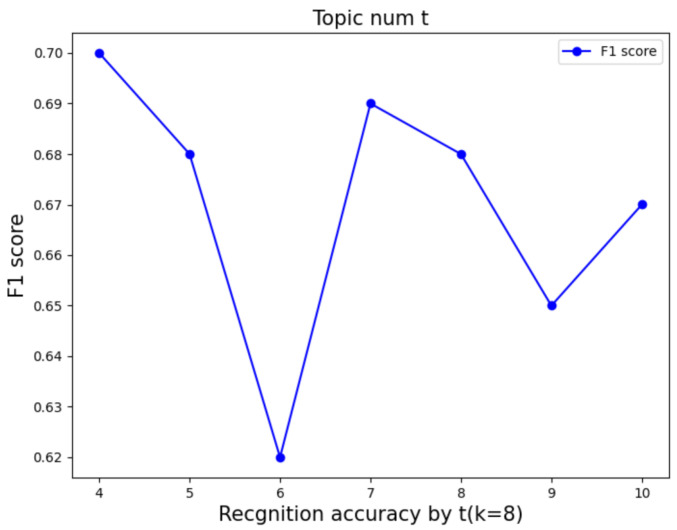
Parameter tuning of topics number of LDA.

The performance of transforming the features of multiple active segments of PD patients into a document–word matrix primarily depends on the number of cluster centers in k-means. A greater number of k-means cluster centers results in a richer variety of words. To identify the optimal number of cluster centers for word types, we set the number of cluster centers as k∈ [4, 5, 6, 7, 8, 9, 10, 11, 12, 13, 14, 15, 16, 17]. Given the limited number of samples in the real PD dataset, we refrained from adding more cluster centers as it could potentially increase word types, making model classification more challenging. Additionally, another crucial hyperparameter is the number of topics in the LDA model as it affects the interpretability of the LDA model on documents and feature extraction. We set the number of topics t∈ [4, 5, 6, 7, 8, 9, 10] to evaluate the impact of different topic numbers on the model. Figure 9 and Figure 10 display the F1-score under various parameter settings. Ultimately, we selected the parameter configuration with cluster center k=8 and topic number t=4, as it achieved the best accuracy at 71.08%.

### 5.5. Experimental Results

**Figure 11:**
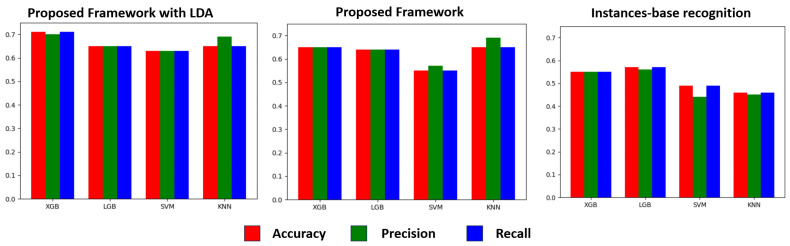
Performance results of real PD dataset.

Experiment 1: In this experiment, we utilized four machine learning models to report the classification results of four stages of PD based on multiple patient activities: XGBoost (XGB), LightGBM (LGBM), the k-nearest neighbor algorithm (KNN), and support vector machine (SVM). Figure 11 displays the performance of our framework and the baseline based on instance recognition in terms of precision, recall, and accuracy. It is evident that XGB exhibits superior classification performance, and our proposed framework, after incorporating LDA features, shows significant improvement (achieving a peak accuracy rate of 71.08%, which is 16% higher than the instance-based method and 6% higher than the MIL method without LDA features), demonstrating the effectiveness of our proposed framework. Table 6 shows the ablation experiments of different methods. It can be seen that the accuracy after adding LDA features is higher than the original method. In subsequent data augmentation research, we selected XGB as our classification model for comparison with other sample generation methods. In Table 7, we compare eight different Multi-Instance Learning methods. It can be seen that for PD diagnosis, our framework is higher than the other basic Multi-Instance Learning methods, indicating that our method is more effective in free-environment PD. There is an improvement effect under weak supervision settings. We speculate that this mapping method takes into account the correlation between instances in a bag, thereby achieving better accuracy. PD-severity assessment needs to consider multiple instances of activity.

Experiment 2: Table 8 presents the XGBoost classification performance after adding data augmentation components to our framework. Additionally, we compared common imbalanced sampling methods and data enhancement techniques, including random oversampling, RandomUnderSample, SMOTE, SMOTETomek, and ADASYN. Notably, among all the methods the hybrid method employing similar pairs achieved the best classification performance, reaching 73.48%, which is 2.4% higher than before data augmentation. Our proposed method effectively increases data diversity while keeping features consistent, thereby enhancing the classification performance of minority classes.

## 6. Conclusions

In this study, we aimed to assess PD stages using a single wearable sensor attached to the right hand. We conducted our research on a real dataset comprising 85 individuals with PD. During our investigation, we identified two key challenges: weak labeling and data imbalance. To address these issues, we introduced a framework for PD-stage evaluation represented by symbols. We employed topic modeling to enhance feature representation within this framework. Additionally, we incorporated a data augmentation component to diversify our dataset, exploring various sample generation techniques such as SMOTE, ADASYN, and SMOTETomek, among others. Furthermore, we introduced a novel similarity-based pattern mixing method. As a result, our final model achieved an impressive accuracy rate of 73.48%. This demonstrates the framework’s ability to mitigate the impact of weak annotations and to enhance data diversity. In summary, our research contributes to more accurate self-diagnosis of PD in real-world settings, offering the potential for remote guidance on medical interventions by healthcare professionals.

In future work, we will continue to dig deeper into the performance of using wearable sensors to accurately assess the severity of PD conditions. In this work, we only independently analyze and utilize the sensor data corresponding to a single activity. Considering the inevitable connection between multiple activities performed by each patient, a more natural and promising approach is to use multi-task learning [40,41] to fully mining the complex relationship between multiple activities, so as to utilize the inherent shared information among multiple activities, further improving the predictive performance and robustness of the model.

## Figures and Tables

**Table 1:** Demographic data of study population (mean and std. stand for mean value and standard deviation, respectively).

	Healthy	PD	Total
Number of patients	15	70	85
Age (mean ± std.)	23.56 ± 2.24	67.57 ± 7.84	49.78 ± 20.47
Weight (mean ± std.)	62.42 ± 5.52	58.51 ± 9.60	62.48 ± 10.24
Height (mean ± std.)	171.66 ± 11.39	160.51 ± 7.73	169.25 ± 9.05
UPDRS level	Healthy: 15	Mild: 41	/
		Moderate: 17	
		Severe: 12	
Gender	Male: 13	Male: 37	Male: 50
	Female: 2	Female: 33	Female: 35
Number of instances	1618	7821	9439

**Table 2:** Detailed information about the 12 activities, including activity name and description.

NO.	Activity Name	Activity Description
1	Finger taps	Quickly pinch the thumb and index finger of both hands.
2	Clenching and opening alternately	Make a fist and open it quickly.
3	Rapid alternating movements of hands	Quickly flip both hands at the same time.
4–5	Hand rotation—right/left	Continuously pat left/right palm with the palm of the right/left hand and the back of the hand.
6–7	Finger to nose—left/right	Touch the tip of nose with left/right index finger, then touch the doctor’s index finger and repeat.
8	Standing with arms held out	Raise your hands and stand with them at shoulder height for 30 s.
9	Walking back and forth	Complete 10 m of walking in a straight line, turn and walk in opposite direction.
10	Rising from chair	Cross your arms on your chest and stand up from the chair.
11	Drinking water	Pick up a cup from the table to drink water.
12	Picking things up	Pick up paper from the floor.

**Table 3:** Parameter settings of traditional machine learning algorithms.

Algorithm	Parameter	Value
KNN	Weights	Uniform
	N neighbors	5
	Distance	Minkowski
XGB	Learning rate	0.3
	N estimators	650
	Max-depth	3
SVM	Kernel	Poly
	Penalty C	1
LightGBM	Learning rate	0.1
	N estimators	500
	Max-depth	2

**Table 4:** Accuracy using a single activity instance.

Activity No.	Method (Accuracy, %)				
	KNN	XGB	SVM	LightGBM	Average
1	38.82	42.35	49.41	37.65	42.05
2	42.35	49.41	45.88	41.18	44.70
3	48.15	49.38	51.85	51.85	50.30
4	51.81	53.01	53.01	46.99	51.20
5	38.55	48.19	51.81	50.60	47.28
6	44.44	41.98	48.15	37.04	42.90
7	45.78	46.99	46.99	45.78	46.38
8	34.52	55.95	50.00	51.19	47.91
9	46.99	46.99	45.78	39.76	44.88
10	35.14	50.00	51.35	52.70	47.29
11	50.00	51.25	50.00	51.52	50.69
12	29.58	47.98	53.52	50.07	45.28

**Table 5:** PD-assessment accuracy of multi-activity bags.

Activity Bags	Method (Accuracy, %)				
	KNN	XGB	SVM	LightGBM	Average
3 + 8	26.83	54.88	47.56	47.56	44.20
3 + 4	55.42	63.86	44.58	68.67	58.13
4 + 8	51.81	63.86	56.63	60.24	58.13
3 + 2 + 4	57.83	56.63	59.04	66.27	59.94
**3 + 2 + 4 + 5**	**65.06**	**71.08**	**62.65**	**65.06**	**65.96**
2 + 3 + 4 + 5 + 7	57.32	59.76	65.85	58.54	60.36
2 + 3+ 4 + 5 + 8	48.78	69.51	57.32	62.20	59.45
2 + 3 + 4 + 5 + 7 + 8	39.51	70.37	56.79	70.37	59.26

**Table 6:** Ablation experiments of the proposed framework.

Method	Accuracy (%)			
	KNN	XGBoost	SVM	LightGBM
Instance Base	46.36	55.88	49.22	56.75
Proposed Framework	65.06	65.06	55.42	63.86
**Proposed Framework with LDA**	**65.06**	**71.08**	**62.65**	**65.06**

**Table 7:** Weakly supervised learning results.

Bag-Mapping Method	Accuracy	Precision	Recall	F1-Score
FV	65.06	64.78	65.06	64.19
MILIBRT	65.06	64.49	65.06	64.41
MIBRV	56.63	58.40	56.63	54.83
MILES	68.67	68.41	68.67	67.42
MILWA	55.88	55.19	55.88	55.11
SimpleMI (median)	66.27	65.87	66.27	65.72
SimpleMI (mean)	65.06	64.87	65.06	64.90
SimpleMI (min–max)	63.86	62.95	63.86	63.14
BOF	57.83	57.47	57.83	57.25
**Ours**	**71.08**	**70.98**	**71.08**	**70.17**

**Table 8:** Data augmentation results.

Method	Accuracy	Precision	Recall	F1-Score
No augmentation	71.08	70.98	71.08	70.17
**Bag augmentation**	**73.48**	**73.68**	**73.48**	**72.93**
SMOTETomek	68.67	68.54	68.67	68.01
ADASYN	66.27	65.70	66.27	65.75
RandomUnderSample	51.81	53.86	51.81	50.10
RandomOverSample	72.29	72.31	72.29	71.85
SMOTE	67.47	67.35	67.47	67.02

## Data Availability

All the data during the current study are provided by AntData Ltd. and go through ethical approval. The data are available from the corresponding author (Po Yang) on reasonable request.
